# Prognostic impact of persistent lower neutrophil-to-lymphocyte ratio during preoperative chemoradiotherapy in locally advanced rectal cancer patients: A propensity score matching analysis

**DOI:** 10.1371/journal.pone.0214415

**Published:** 2019-03-22

**Authors:** Yoon Jin Cha, Eun Jung Park, Seung Hyuk Baik, Kang Young Lee, Jeonghyun Kang

**Affiliations:** 1 Department of Pathology, Gangnam Severance Hospital, Yonsei University College of Medicine, Seoul, South Korea; 2 Department of Surgery, Gangnam Severance Hospital, Yonsei University College of Medicine, Seoul, South Korea; 3 Department of Surgery, Severance Hospital, Yonsei University College of Medicine, Seoul, South Korea; University of South Alabama Mitchell Cancer Institute, UNITED STATES

## Abstract

**Purpose:**

This study investigated the significance of change in neutrophil-to-lymphocyte ratio (NLR) during preoperative chemoradiotherapy (preop-CRT) in patients with non-metastatic rectal cancer using a propensity score matching method (PSM).

**Methods:**

Patients who underwent surgery after completion of preop-CRT for non-metastatic rectal cancers from Jan 2004 to Dec 2013 were retrospectively enrolled. NLRs were obtained before commencement of CRT (pre-NLR) and between completion of CRT and surgery (post-NLR). Using Cox regression hazards models, the association of NLRs with survival after PSM was examined.

**Results:**

A total of 131 patients were grouped as follows: group A, pre-NLR < 3 & post-NLR < 3 (n = 47); group B, pre-NLR < 3 & post-NLR ≥ 3 (n = 45); group C, pre-NLR ≥ 3 & post-NLR < 3 (n = 5); group D, pre-NLR ≥ 3 & post-NLR ≥ 3 (n = 34). There was no difference in disease-free survival (DFS) or overall survival (OS) rate according to group. When dichotomized into group A versus groups B-D, DFS was higher in group A (84.7%) than groups B-D (67.5%, p = 0.021). After PSM (n = 94), multivariable analysis identified persistent lower NLR as an independent favorable prognosticator of DFS (HR 0.37, 95% CI 0.15–0.92, p = 0.033).

**Conclusions:**

Persistent non-inflammatory state measured by NLR may be an indicator of decreased risk of recurrence in patients with locally advanced rectal cancer treated with preop-CRT.

## Introduction

Colorectal cancer is a major cause of cancer-related death worldwide and in South Korea.[1–3] Among them, nearly 30% of patients had been diagnosed with rectal cancer.[2] According to guidelines, standard care for locally advanced rectal cancer (clinical stage II and III) is preoperative chemoradiotherapy (preop-CRT) followed by total mesorectal excision.[4,5] Although preop-CRT may decrease local recurrence rate in comparison to postoperative adjuvant chemoradiotherapy, it does not improve overall survival (OS).[4,5] Risk stratification for high risk patients to designate proper management is important to improve prognostic outcomes in these patients.

Various markers reflecting systemic inflammatory response, such as neutrophil-to-lymphocyte ratio (NLR), platelet-to-lymphocyte ratio (PLR), lymphocyte-to-monocyte ratio (LMR), C-reactive protein (CRP), and the modified Glasgow prognostic score (mGPS), are demonstrated prognostic factors for survival in primary operable cancers.[6] Although albumin or CRP are not usually assessed as part of the preoperative workup for cancer patients, differential white cell count is routinely performed to identify patients with hypercoagulability or infection risk.[6] Among these laboratory markers, NLR is one of the most commonly used biomarkers. It was reported that tumor affected the hematopoietic progenitor cell of the host and thus myeloid lineage populations (including neutrophils) could expand.[7] These myeloid-derived suppressor cells (MDSCs) suppress host immune cells through various pathways and could diminish lymphocytes.[8] NLR could be used to estimate the relative balance of myeloid and lymphocytic lineages, thus reflecting host immunity in cancer patients.[8] The prognostic impact of NLR was thoroughly investigated in different types of diseases including gastric cancer, hepatocellular cancer, pancreatic cancer, head and neck cancer, esophageal cancer, breast cancer, and thyroid cancer.[9–15] In accordance with these results, previous studies including a meta-analysis demonstrated that NLR can predict survival outcome,[16–22] or can also predict tumor regression grade, such as pathologic complete response (pCR), or good tumor response after preop-CRT in patients with rectal cancer.[18,20,23–25]

Nevertheless, there are also a number of studies that have shown that NLR is not relevant in patients with rectal cancer. Lino-Silva and colleagues reported that there were no differences in survival outcomes or pCR rate according to NLR cut-off point (2.0, 2.5, 4, and 5) in 175 patients who underwent preop-CRT.[26] Shen and colleagues also reported that NLR measured in pre-CRT did not predict OS or disease-free survival (DFS) in patients with locally advanced rectal cancer who underwent preop-CRT.[27] Jung et al. reported that NLR measured before commencement of preop-CRT could not discriminate recurrence-free survival (p = 0.07) among 984 patients who underwent preop-CRT.[28] Portale and coworker recently demonstrated that neither PLR nor NLR were associated with survival and recurrence in patients undergoing laparoscopic curative resection for rectal cancer with or without preop-CRT.[29] The basis of this discrepancy among studies is not clearly understood.

The long course of preop-CRT for locally advanced rectal cancer patients usually takes 11–15 weeks from preop-CRT commencement to the date of definite surgery. As has been recently suggested, neutrophil and lymphocyte counts are not constant over time during preop-CRT for rectal cancer.[30] So, the NLR will vary in value. Most previous studies evaluating the impact of NLR on survival or tumor response for rectal cancer measured NLR at a specific time, mainly before initiating preop-CRT, or used the NLR value assessed before preop-CRT.[16–20,23–29,31] In contrast, the prognostic significance of change in NLR during preop-CRT for rectal cancer has not been widely assessed.[32]

Our study aimed to investigate the impact of changes in pre- and post-chemoradiotherapy neutrophil-to-lymphocyte ratios (NLRs) on the prognosis of patients with rectal cancer who underwent preop-CRT.

## Materials and methods

A retrospective cohort study was conducted using consecutive patients with non-metastatic rectal cancer who underwent preop-CRT followed by surgery from Jan 2004 to Dec 2013. All patients diagnosed with clinical stage II or III rectal cancer who underwent long-course preop-CRT were screened. Patients who underwent emergent or palliative surgeries, were diagnosed as stage IV at initial staging workup, with history of inflammatory bowel disease, or with any missing blood examination data during preop-CRT were excluded from this study. Finally, 131 patients were included in this analysis.

Routine demographic variables, surgical outcomes, and oncologic outcomes were obtained from the electronic medical records, including age, sex, body mass index (BMI), carcinoembryonic antigen (CEA), neutrophil, lymphocyte, monocyte and platelet counts, type of surgery, hospital stay, recurrence, and survival. NLR was defined by dividing absolute neutrophil count by absolute lymphocyte count and was calculated twice for each patient using the complete blood count (CBC) performed before CRT (pre-NLR) and between completion of CRT and surgery (post-NLR). Additional blood test between these two examinations was not routinely performed. This study was approved by Gangnam Severance Hospital’s Institutional Review Board. Informed consent was waived for this retrospective study.

### Preoperative chemoradiotherapy, total mesorectal excision and follow up

All patients underwent conventional radiotherapy (RT) with concurrent 5-fluorouracil-based chemotherapy. Mean 50.4 Gy radiation dose was irradiated in 28 fractions over 5 weeks. RT was applied to the whole pelvis with 45 Gy in 25 fractions, with a boost of 5.4 Gy to the primary tumor in 3 fractions. Patients were treated with 3 portals of posterior and bilateral beams in the prone position. The superior border was 1.5 cm above the sacral promontory (L5 level), and the inferior border was at the inferior margin of the obturator foramen or 3 cm below the lower tumor margin. The lateral border was 1.5 cm lateral to the bony pelvis, and the anterior border was 3 cm anterior to the tumor. The posterior border was 0.5 cm posterior to the sacral surface. The boost volume was 3 cm expansion from the primary tumor in the superior and inferior directions and 2 cm expansion radially. Chemotherapy regimens were either intravenous 5-fluorouracil (5-FU) or oral capecitabine. Intravenous chemotherapy was administered at a dose of 425 mg/m^2^/day of 5-FU with 20 mg/m^2^/day of leucovorin during the first and fifth weeks of radiation treatment (RT). Oral capecitabine was administered at a dose of 1,650 mg/m^2^/day during the whole RT period. Surgery was performed in accordance with the principle of total mesorectal excision (TME) at 6 to 12 weeks after completion of preop-CRT. After surgery, patients were followed up in an out-patient clinic every 3 months for the first 3 years and 3–6 months thereafter until 5 years from the initial surgery. CEA was assessed at each visit. Chest and abdominopelvic computed tomography (CT) was performed every 6 or 12 months according to postoperative stage. Colonoscopy was recommended at 1, 3, and 5 years after surgery. Positron-emission tomography scan or magnetic resonance imaging was added according to physician discretion. Local and distant metastases were defined according to clinical and radiologic evaluations. Biopsy confirmation was not always mandatory to confirm recurrence.

### Classifications according to NLRs measured at two different time points during preoperative chemoradiotherapy

To assess the significance of the change in NLRs while receiving preop-CRT, patients were stratified into 4 groups using the cut-off value of 3 for both pre-NLR and post-NLR. This cut-off value was derived from previous studies on the impact of NLR for patients with colorectal cancer.[33–35] In brief, patients were grouped as follows: group A, pre-NLR < 3 & post-NLR < 3; group B, pre-NLR < 3 & post-NLR ≥ 3; group C, pre-NLR ≥ 3 & post-NLR < 3; group D, pre-NLR ≥ 3 & post-NLR ≥ 3. Patients were further sub-stratified as group A versus groups B-D based on survival outcomes in further analysis.

### Propensity score matching

After sub-stratifying the entire cohort into group A versus groups B-D, significant difference in clinical T stage and marginal difference in clinical N stage was identified between the two groups, which might affect long-term oncologic outcomes. The following covariates were included in the model to calculate the propensity score: clinical T stage and clinical N stage. The NLR dichotomization (group A vs. control) was entered into the regression model as the dependent variable. Matching of propensity scores was obtained with the 1:1 optimal matching method.

### Further analysis of the impact of neutrophils, lymphocytes, PLR and LMR on DFS and OS

Using the cohort after propensity score matching, we further evaluated the significance of neutrophil, lymphocyte, PLR and LMR with DFS and OS. PLR was defined as the absolute platelet count divided by the absolute lymphocyte count. LMR was defined as the absolute lymphocyte count divided by the absolute monocyte count. First, patients were stratified into the 2 groups using the cut-off value of median for neutrophils, lymphocytes, PLR and LMR respectively. Second, patients were separately into four grouped as follows: pre- < median & post- < median; pre- < median & post- ≥ median; pre- ≥ median & post- < median; pre- ≥ median & post- ≥ median. Finally, patients were sub-stratified as pre- < median & post- < median vs. control in further analysis.

### Statistical analysis

Differences in clinicopathologic features between groups were analyzed using the chi-square test or Fisher’s exact test for categorical variables and with Student’s t-test for continuous variables. The primary clinical outcomes of interest were OS and DFS. OS duration was defined as the time from the date of operation to the date of death or last follow-up. DFS duration was defined as time from the date of operation to the date of recurrence (local recurrence or/and distant metastasis), death, or last follow-up. Local recurrence-free survival (LRFS) and distant metastasis-free survival (DMFS) were defined as time from the date of surgery to the occurrence of locoregional recurrence or distant metastasis, respectively. The Kaplan–Meier method was used for survival analysis, and the log-rank test was used to compare survival outcomes between the groups. Univariable and multivariable analyses were performed using a Cox proportional hazards model. Variables that had significance of p ≤ 0.1 on univariable analysis were eligible for inclusion in multivariable analysis. Multivariable models were derived using forward stepwise selection. A two-sided p < 0.05 was considered statistically significant. Statistical analyses were performed using IBM SPSS version 23.0 (IBM Corp., Armonk, NY, USA) and R version 3.5.1 (R-project, Institute for Statistics and Mathematics).

## Results

### Patient characteristics

A total of 131 patients were included in this study. The median follow-up period was 73.3 [interquartile range (IQR) 56.2–98.1)] months. The majority of patients was male (65.6%) and diagnosed with low rectal tumor (tumor was located below 6 cm from the anal verge) (71.8%). The median age was 59 (IQR, 51–67) years, and median BMI was 23.3 (IQR, 21.4–24.8) kg/m^2^. Median CEA before starting preop-CRT was 4 (IQR, 2–8) ng/mL. In pre-treatment staging before preop-CRT, there were 19 (14.5%), 67 (51.1%), and 45 (34.4%) cT2, cT3, and cT4, respectively. Most patients (80.2%) were diagnosed as clinically node positive.

The median values of pre-NLR and post-NLR were 2.25 (IQR, 1.64–3.29) and 3.46 (IQR, 2.57–4.75), respectively (p < 0.01). Median days from measurement of pre-NLR and post-NLR to the date of surgery were 98 (IQR, 84–109) and 13 (IQR, 7–18) days, respectively. The proportion of patients with greater than 3 NLR was 29.8% in pre-NLR and 60.3% in post-NLR (p < 0.001). Combining the cut-off value of 3 in pre- and post-NLRs, 47 (35.9%), 45 (34.4%), 5 (3.8%), and 34 (26%) patients were classified into group A, group B, group C, and group D, respectively (Table 1).

**Table 1:** Clinicopathologic characteristics of overall patients (n = 131).

		N (%)
Gender	Male	86 (65.6)
	Female	45 (34.4)
Age (years)	median (IQR^a^)	59 (51–67)
BMI^b^ (kg/m^2^)	median (IQR)	23.3 (21.4–24.8)
Tumor distance from anal verge (cm)	< 6	94 (71.8)
	≥ 6	37 (28.2)
Pre-CRT CEA^c^ (ng/mL)	median (IQR)	4 (2–8)
cT stage	cT2	19 (14.5)
	cT3	67 (51.1)
	cT4	45 (34.4)
cN stage	cN (–)	26 (19.8)
	cN (+)	105 (80.2)
pre-NLR	median (IQR)	2.25 (1.64–3.29)
	< 3	92 (70.2)
	≥ 3	39 (29.8)
post-NLR	median (IQR)	3.46 (2.57–4.75)
	< 3	52 (39.7)
	≥ 3	79 (60.3)
Combination of pre&post NLRs	pre-NLR<3 & post-NLR<3 (Group A)	47 (35.9)
	pre-NLR<3 & post-NLR≥3 (Group B)	45 (34.4)
	pre-NLR≥3 & post-NLR<3 (Group C)	5 (3.8)
	pre-NLR≥3 & post-NLR≥3 (Group D)	34 (26)

^a^IQR: interquartile range

^b^BMI: body mass index

^c^CEA: carcinoembryonic antigen

### Overall survival, disease-free survival, local recurrence-free survival, and distant metastasis-free survival among 131 patients

Of the 131 patients, local recurrence was observed in 6 (4.5%) and distant metastasis occurred in 30 (22.9%) during the study period. Overall, death occurred in 38 patients (29%). The 5-year OS and DFS rates were 80.1% and 73.6%, respectively, for all patients. The 5-year LRFS and DMFS were 95.3% and 78%, respectively, for all patients.

**Fig 1:**
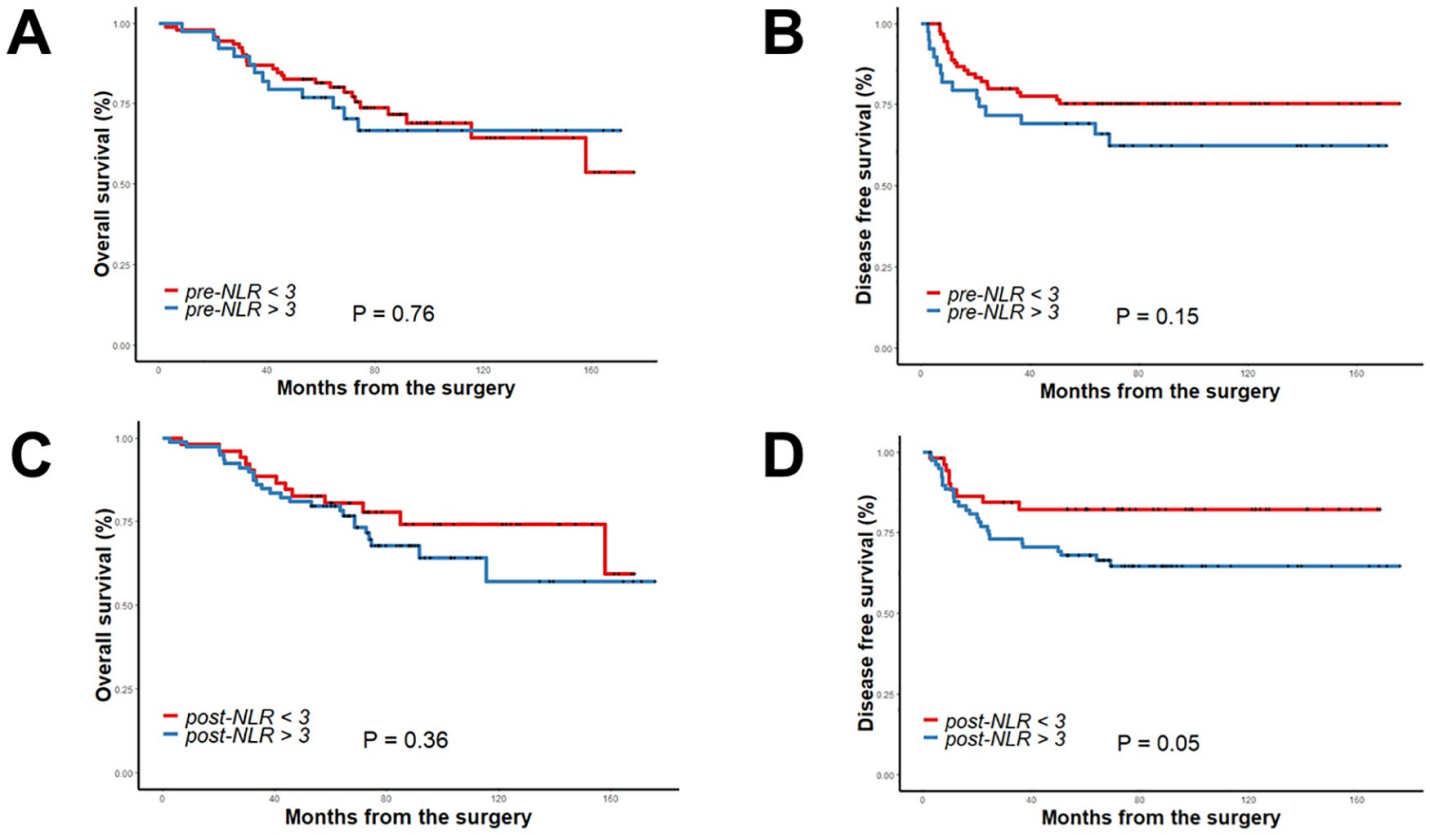
Overall survival and disease-free survival according to cut-off value 3 of pre-NLR (A, B) and post-NLR (C, D).

When survival outcomes were compared according to the cut-off value of 3 in pre-NLR, OS and DFS did not differ between the pre-NLR < 3 and pre-NLR ≥ 3 groups (OS: p = 0.76, DFS: p = 0.15). At a cut-off value of 3 for post-NLR, there was no difference of OS (p = 0.36) between the post-NLR < 3 and post-NLR ≥ 3 groups. There was a trend toward better DFS in the post-NLR < 3 group compared with the post-NLR ≥ 3 group (p = 0.05) (Fig 1A–1D).

**Fig 2:**
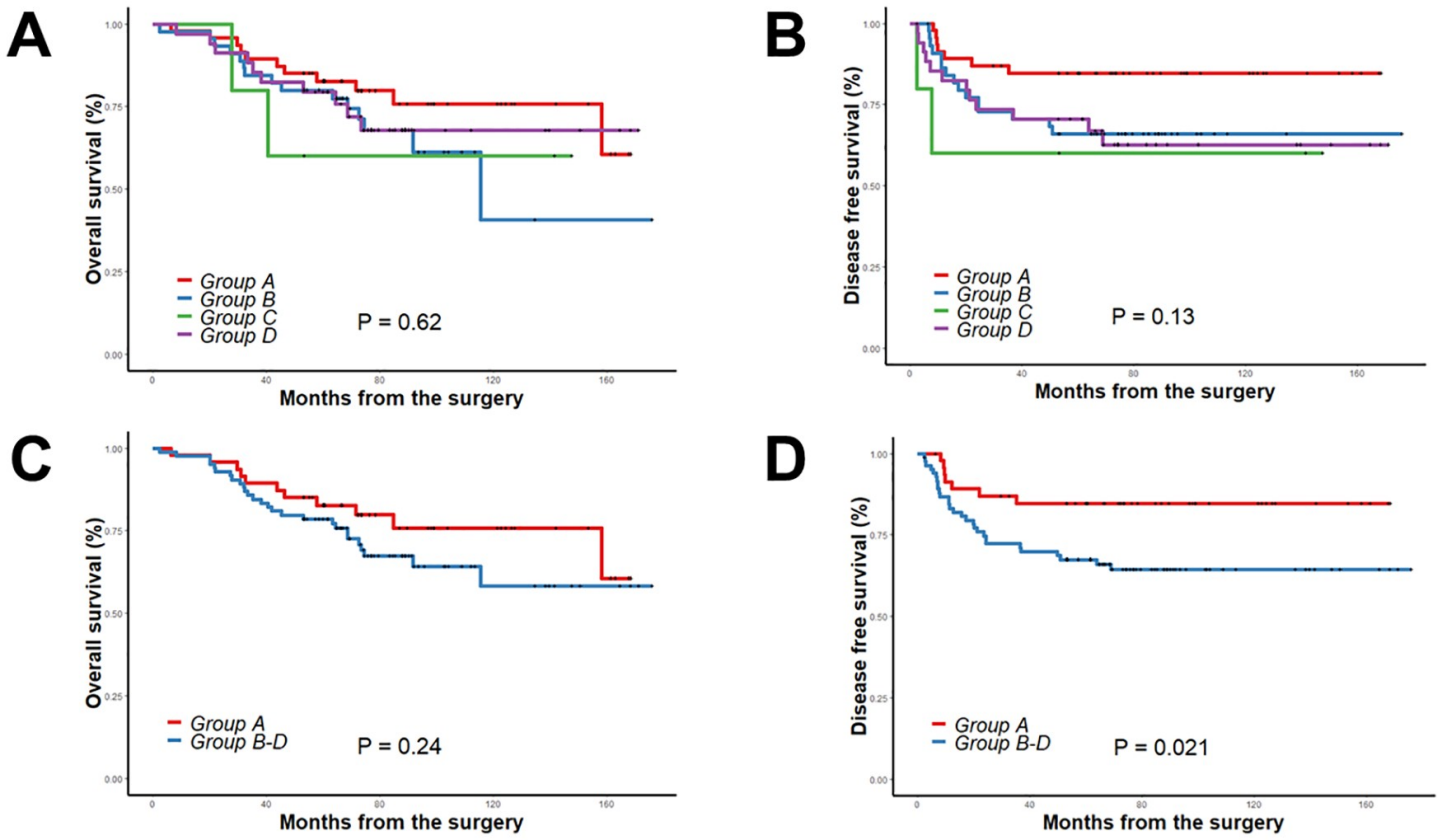
Overall survival and disease-free survival according to combination of pre and post NLRs using cut-off value 3 (A, B) and according to group A versus groups B-D (C, D) in whole cohort (n = 131).

Next, OS and DFS were compared among the 4 groups (groups A-D). There was no significant difference in 5-year OS or 5-year DFS among the four groups (OS: 82.7% in group A, 80% in group B, 60% in group C, and 79.4% in group D, p = 0.620, DFS: 84.7% in group A, 65.9% in group B, 60% in group C, and 70.6% in group D, p = 0.125) (Fig 2A and 2B). When patients were further sub-stratified into group A versus groups B-D, there was no significant difference in 5-year OS between the two groups (82.7% in group A vs. 78.6% in groups B-D, p = 0.245). In contrast, the 5-year DFS rate was significantly better in group A than in groups B-D (84.7% in group A vs. 67.5% in groups B-D, p = 0.021) (Fig 2C and 2D).

### Propensity score matching

The patient characteristics of group A versus groups B-D are described in Table 2. Among 131 patients (before PSM), a significantly higher proportion in groups B-D had advanced clinical T stage (p = 0.008). Although it was not statistically significant, clinically node positive patients were more predominant in groups B-D compared to group A (84.5% vs. 72.3%, p = 0.112). Because this imbalance of preoperative clinical staging could impact survival outcomes, PSM with the 1:1 matching method was performed using clinical T and N stages. After matching, there was no difference in clinicopathologic variables between the two groups.

**Table 2:** Patients’ characteristics according to combination of pre and post-chemoradiotherapy neutrophil-to-lymphocyte ratio before and after propensity score matching (PSM).

		Unmatched patients (n = 131)			Matched patients (n = 94)		
		Group A (n = 47) n (%)	Group B-D (n = 84) n (%)	P	Group A (n = 47) n (%)	Control (n = 47) n (%)	P
Gender	Male	28 (59.6)	58 (69)	0.338	28 (59.6)	33 (70.2)	0.388
	Female	19 (40.4)	26 (31)		19 (40.4)	14 (29.8)	
Age (years)	< 65	31 (66)	62 (73.8)	0.423	31 (66)	35 (74.5)	0.499
	≥ 65	16 (34)	22 (26.2)		16 (34)	12 (25.5)	
BMI^a^ (kg/m^2^)	< 25	34 (72.3)	68 (81)	0.278	34 (72.3)	35 (74.5)	1.0
	≥ 25	13 (27.7)	16 (19)		13 (27.7)	12 (25.5)	
Tumor distance from anal verge (cm)	< 6	40 (85.1)	54 (64.3)	0.015	40 (85.1)	38 (80.9)	0.785
	≥ 6	7 (14.9)	30 (35.7)		7 (14.9)	9 (19.1)	
Pre-CRT CEA^b^ (ng/mL)	< 5	33 (70.2)	48 (57.1)	0.189	33 (70.2)	28 (59.6)	0.388
	≥ 5	14 (29.8)	36 (42.9)		14 (29.8)	19 (40.4)	
cT stage^c^	cT2	11 (23.4)	8 (9.5)	0.008	11 (23.4)	8 (17)	0.772
	cT3	27 (57.4)	40 (47.6)		27 (57.4)	30 (63.8)	
	cT4	9 (19.1)	36 (42.9)		9 (19.1)	9 (19.1)	
cN stage^c^	cN (–)	13 (27.7)	13 (15.5)	0.112	13 (27.7)	10 (21.3)	0.632
	cN (+)	34 (72.3)	71 (84.5)		34 (72.3)	37 (78.7)	
Operation method	LAR^d^	25 (53.2)	43 (51.2)	0.890	25 (53.2)	17 (36.2)	0.241^e^
	CAA^f^ /ISR^g^	19 (40.4)	33 (39.3)		19 (40.4)	25 (53.2)	
	APR^h^/Hartmann	3 (6.4)	8 (9.5)		3 (6.4)	5 (10.6)	
Complications	Yes	16 (34)	31 (36.9)	0.850	16 (34)	15 (31.9)	1.0
	No	31 (66)	32 (68.1)		31 (66)	32 (68.1)	
Anastomotic leakage^i^	Yes	1 (2.3)	4 (5.3)	0.651	1 (2.3)	0	1.0
	No	43 (97.7)	72 (94.7)		43 (97.7)	42 (100)	
Tumor size (cm)	< 5	47 (100)	71 (93.4)	0.157^e^	47 (100)	45 (95.7)	0.495^e^
	≥ 5	0	5 (6.6)		0	2 (4.3)	
ypT	ypT0-2	31 (66)	35 (41.7)	0.011	31 (66)	23 (48.9)	0.144
	ypT3-4	16 (34)	49 (58.3)		16 (34)	24 (51.1)	
ypN	Negative	38 (80.9)	57 (67.9)	0.153	38 (80.9)	36 (76.6)	0.802
	Positive	9 (19.1)	27 (32.1)		9 (19.1)	11 (23.4)	
pCR^j^	Yes	10 (21.3)	12 (14.3)	0.336	10 (21.3)	7 (14.9)	0.593
	No	37 (78.7)	72 (85.7)		37 (78.7)	40 (85.1)	
CRM^k^	Positive (≤ 1 mm)	1 (2.1)	3 (3.9)	0.834^e^	1 (2.1)	2 (4.3)	0.164^e^
	Negative (> 1 mm)	17 (36.2)	31 (40.8)		17 (36.2)	9 (19.1)	
	missing	29 (61.7)	42 (55.3)		29 (61.7)	36 (76.6)	
Postoperative chemotherapy	None	8 (17)	11 (13.1)	0.519^e^	8 (17)	4 (8.5)	0.587^e^
	IV 5FU^l^ / Oral 5FU	37 (78.7)	65 (77.4)		37 (78.7)	40 (85.1)	
	FOLFOX^m^ / FOLFIRI^n^	2 (4.3)	8 (9.5)		2 (4.3)	3 (6.4)	

^a^BMI: body mass index

^b^CEA: carcinoembryonic antigen

^c^: Matched variables

^d^LAR: low anterior resection

^e^: Fisher’s exact test

^f^CAA: coloanal anastomosis

^g^ISR: intersphincteric resection

^h^APR: abdominoperineal resection

^i^: Of the 120 and 86 patients respectively who underwent sphincter preserving procedures (low anterior resection or coloanal anastomosis/intersphincteric resection)

^j^pCR: pathologic complete response

^k^CRM: circumferential resection margin

^l^FU: 5-fluorouracil/leucovorin

^m^FOLFOX: folinic acid, 5-fluorouracil, oxaliplatin

^n^FOLFIRI: folinic acid, 5-fluorouracil, irinotecan

### Univariable and multivariable analyses for DFS after PSM

Using the selected cohort after PSM (n = 94), univariable and multivariable analyses were performed for DFS. In univariable analysis, pre-CRT CEA, tumor distance from the anal verge, tumor size, ypT, ypN, and combination of pre- and post-NLRs were identified as significant risk factors for DFS. In multivariable analysis, ypT3-4 vs. ypT0-2 [Hazard Ratio (HR) 2.42, 95% confidence interval (CI) 1–5.8, p = 0.048], yp node positive vs. yp node negative (HR 5.6, 95% CI 2.43–12.93, p < 0.001), and pre-NLR<3 & post-NLR<3 vs. control (HR 0.37, 95% CI 0.15–0.92, p = 0.033) remained as independent prognostic factors for DFS (Table 3).

**Table 3:** Univariable and multivariable Cox-regression analysis for DFS after PSM (n = 94).

		Univariable analysis		Multivariable analysis	
		Hazard Ratio (95% CI)	P	Hazard Ratio (95% CI)	P
Gender	Male	1			
	Female	0.9 (0.38–2.11)	0.820		
Age (years)	< 65	1			
	≥ 65	1 (0.43–2.51)	0.925		
BMI^a^ (kg/m^2^)	< 25	1			
	≥ 25	0.67 (0.25–1.81)	0.437		
cT stage	cT2	1			
	cT3	2.82 (0.64–12.27)	0.167		
	cT4	3.89 (0.78–19.28)	0.096		
cN stage	cN (–)	1			
	cN (+)	4.05 (0.95–17.23)	0.058		
Pre-CRT CEA^b^ (ng/mL)	< 5	1			
	≥ 5	2.3 (1.0–5.14)	0.042		
Tumor distance from anal verge (cm)	< 6	1			
	≥ 6	2.49 (1.03–6.04)	0.042		
Operation name	LAR^c^	1			
	CAA^d^ & ISR^e^	0.55 (0.22–1.32)	0.185		
	APR^f^ & Hartmann	1.26 (0.36–4.43)	0.715		
Operation time (min)	< 360	1			
	≥ 360	1.31 (0.49–3.53)	0.582		
Tumor size (cm)	< 5	1			
	≥ 5	5.28 (1.23–22.7)	0.025		
ypT	ypT0-2	1		1	
	ypT3-4	3.26 (1.39–7.63)	0.006	2.42 (1–5.8)	0.048
ypN	Negative	1		1	
	Positive	5.88 (2.62–13.19)	<0.001	5.6 (2.43–12.93)	<0.001
CRM^g^	Positive (≤ 1 mm)	1			
	Negative (> 1mm)	1.39 (0.18–10.7)	0.746		
	missing	1.31 (0.17–9.76)	0.792		
Complications	No	1			
	Yes	2.05 (0.92–4.6)	0.079		
pre-NLR	< 3	1			
	≥3	2.01 (0.86–4.7)	0.107		
post-NLR	< 3	1			
	≥3	2.08 (0.91–4.77)	0.081		
Combination of pre&post NLRs	Control	1		1	
	pre-NLR<3 & post-NLR<3	0.37 (0.15–0.89)	0.027	0.37 (0.15–0.92)	0.033

^a^BMI: body mass index

^b^CEA: carcinoembryonic antigen

^c^LAR: low anterior resection

^d^CAA: coloanal anastomosis

^e^ISR: intersphincteric resection

^f^APR: abdominoperineal resection

^g^CRM: circumferential resection margin

### Correlations of neutrophil, lymphocytes, PLR and LMR with DFS and OS

The pre- and post- median values of the neutrophil, lymphocytes, PLR and LMR were shown in S1 Table. Using the median values as the cut-off points, prognostic impact of each variable was investigated. There was no survival difference according to neutrophil, lymphocyte, PLR, and LMR either evaluated in pre- and post- measurements (S2 Table). When we classified patients into the four groups using combination of pre- and post- PLRs and LMRs respectively, Kaplan-Meier plot showed no survival difference between the groups (S1 Fig, Fig 1A–1D). Even when we re-classified those four groups into the two groups, such as pre-PLR<154.4 & post-PLR<255.7 vs. control or pre-LMR<5.42 & post-LMR<3.15 vs. control, there was no survival difference between the two groups. When the patients were dichotomized as pre-LMR≥5.42 & post-LMR≥3.15 vs. control, there was no survival difference between the two groups (S3 Table).

## Discussion

This study demonstrated that persistent non-inflammatory status during preop-CRT, assessed by a combination of neutrophil-to-lymphocyte ratios obtained at two different time points, might be associated with low possibility of recurrence in patients with locally advanced non-metastatic rectal cancer who underwent long course preop-CRT followed by surgery.

Previous meta-analysis on the prognostic effect of NLR in patients with rectal cancer showed that elevated NLR was associated with poor survival, with HRs of 13.4 in OS, 4.3 in DFS, and 3.6 in relapse free survival.[22] Nevertheless, several recent studies reported that NLR could not discriminate prognosis in patients with non-metastatic rectal cancer with or without preop-CRT.[26–29] The different impacts of NLR may, to some extent, be explained by weakened predictive power of the NLR due to the narrow spectrum of tumor burden, random error caused by small sample size, or racial differences.[27,29] However, the exact reason for this discrepancy was not clearly depicted. In our study, there was no difference in OS or DFS according to the cut-off value of 3 for pre-NLR. Although DFS was marginally lower in patients with post-NLR > 3, the significance was not sustained in multivariable analysis after propensity score matching. Considering only these results, our study was in line with previous studies reporting the irrelevance of NLR with long-term prognosis in patients with rectal cancer who underwent preop-CRT. However, after combining consecutive values of NLRs during preop-CRT, our cohort can be separated into two distinct groups showing different survival outcomes.

As far as we know, only one study has reported the clinical usefulness of combining pre- and post-NLRs to predict survival outcomes in patients with locally advanced rectal cancer who underwent preop-CRT. Sung and colleagues reported that persistently elevated NLRs measured twice during preop-CRT, in comparison to the persistently lower group, showed poor DFS (HR: 4.35, 95% CI: 1.361–13.901, p = 0.013).[32] It should be mentioned that those authors divided their groups using the cut-off points of 1.75 in pre-CRT NLR and 5.14 in post-CRT NLR, as these values were determined to maximize the log-rank test of survival. However, the cut-off value of 1.75 was relatively lower than that used in previous studies, and most patients (72.5%) were allocated into the high pre-CRT NLR group (Table 4). According to the recent analysis of 12,160 healthy Korean people, the mean ± 1.96 standard deviation of NLR was reported to be 1.65 (0.107–3.193).[36] In addition, mean (95% CI) NLR was reported as 2.15 (2.11–2.19) using 9,427 subjects participating in the National Health and Nutritional Examination Survey in the USA.[37] As those authors already stated, more supporting data are required to accept those cut-off values in clinical practice.

**Table 4:** Results of previous studies on NLR in rectal cancer patients with or without preoperative chemoradiotherapy.

Authors	year/nation	No. of patients (% of CRT)	Measurement	Neutrophil to lymphocyte ratio (NLR)			Correlations	
				Median (range)	Cut-off	High NLR	Long term survival outcomes	Pathologic tumor response
Carruthers et al.[17]	2012/UK	115 (100)	Before CRT	N/A	5.0	N/A	Correlation: (+) Low NLR vs. High NLR: **OS**—HR 7.0, 95% CI (2.6–19.2), ***p = 0*.*006*** **DFS**—HR 4.1, 95% CI (1.7–9.8), ***p = 0*.*03***	Not evaluated
Krauthamer et al.[23]	2013/Israel	71 (100)	Before CRT	N/A	5.0	35.2%	Not evaluated	Correlation: (+) High NLR vs. Low NLR: **pCR—**OR 2.54, 95% CI (1.52–4.18), ***p = 0*.*04*** (Only in clinical stage III patients, multivariable analysis)
Shen L et al.[16]	2014/China	199 (100)	Before CRT	2.4 (1.0–8.9)	2.8	33.2%	Correlation: (+) Low NLR vs. High NLR: **OS**—HR 2.123, 95% CI (1.14–3.954), ***p = 0*.*018*** **DFS**—HR 1.363, 95% CI (0.840–2.214), p = 0.210	Correlation: (-) mean NLR of **TRG 0–1 vs. TRG 2–3**: 2.68 ± 1.42 vs. 2.82 ± 1.33, p = 0.873
Kim IY et al.[18]	2014/Korea	102 (100)	Before CRT	N/A	3.0	24.5%	Correlation: (+) Low NLR vs. High NLR: **Cancer specific survival**—HR 6.6, 95% CI (1.3–32), ***p = 0*.*02* Recurrence free survival**—HR 2.8, 95% CI (1.1–6.8), ***p = 0*.*03***	Correlation: (+) Low NLR vs. High NLR: **poor pathologic tumor response (ypTNM II-IV)—**OR 5.2, 95% CI (1.1–26.5), ***p = 0*.*04***
			After CRT	N/A	3.0	49%	Not evaluated	Correlation: (-) Low NLR vs. High NLR: **poor pathologic tumor response (ypTNM II-IV)—**p = 0.4
Nagasaki et al.[19]	2015/Japan	201(100)	Before CRT	2.3 (0.8–11.1)	3.0	21.9%	Correlation: (+) Low NLR vs. High NLR: **OS**—HR 3.381, 95% CI (1.307–8.751), ***p = 0*.*012*** **RFS**—No association	Not evaluated
Caputo et al.[24]	2016/Italy	87 (100)	Before CRT	2.4 (0.9–9.8)	2.8	35.6%	Not evaluated	Correlation: (-) High NLR vs. Low NLR: **TRG**—p = 0.942
			After CRT	3.7 (1.3–33.4)	3.8	49.4%	Not evaluated	Correlation: (+) High NLR: **Higher rates of TRG 4 response**—***p = 0*.*033***
Hodek et al.[31]	2016/Czech Republic	173 (100)	Before CRT	2.78 (0.64–14.84)	2.8	49.1%	Correlation: (+) Low NLR vs. High NLR: **OS**—***p = 0*.*03*** **DFS**—p = 0.20 (univariable analysis)	Correlation: (-) **pCR**—p = 0.43
Lee et al.[25]	2017/Korea	291 (100)	Before CRT	N/A	5.0	9.6%	Correlation: (+) Low NLR vs. High NLR: **Relapse rate**—OR 2.4, 95% CI (1.09–5.27), ***p = 0*.*025*** (univariable analysis)	Correlation: (+) Low NLR vs. High NLR: **pCR**—15.3% vs. 0%, ***p = 0*.*026*** (univariable analysis)
			After CRT	N/A	5.0	27.1%	Correlation: (-) No detailed data	Correlation: (-) No detailed data
Sung et al.[32]	2017/Korea	110 (100)	Before CRT	2.1 (0.53–10.63)	1.75	72.7%	Correlation: (+) Pre-NLR≤1.75 & Post-NLR≤5.14 vs. Pre-NLR>1.75 & Post-NLR>5.14: **DFS**—HR 4.350, 95% CI (1.361–13.901), ***p = 0*.*013***	Correlation: (-) Pre-NLR≤1.75 & Post-NLR≤5.14 vs. Pre-NLR>1.75 & Post-NLR≤5.14 or Pre-NLR≤1.75 & Post-NLR>5.14 vs. Pre-NLR>1.75 & Post-NLR>5.14: **pCR**—6.9% vs. 13.1% vs. 5%, p = 0.467
			After CRT	3.23 (0.48–21.64)	5.14	19%		
Kim TG et al.[20]	2018/Korea	176 (100)	Before CRT	N/A	2.0	51.7%	Correlation: (+) Low NLR vs. High NLR: **OS**—92.4% vs. 71.9%, p = 0.027 **DFS—**86.8% vs. 70.7%, ***p = 0*.*014*** (multivariable analysis)	Correlation: (+) Low NLR vs. High NLR: **Poor tumor response (Dworak grade 0–2)**—OR 2.49, 95% CI (1.264–4.904), ***p = 0*.*008*** (multivariable analysis)
Vallard et al.[21]	2018/France	257(100)	Before CRT	N/A	2.8	27.6%	Correlation: (+) Low NLR vs. High NLR: **Local recurrence**—OR 14.7, 95% CI (1.53–334.3), ***p = 0*.*03* Progression-free survival**—HR 2.21, 95% CI (1.26–3.86), ***p = 0*.*006*** **Overall survival**—HR 2.23, 95% CI (1.14–2.36), ***p = 0*.*02***	Correlation: (-) Low NLR vs. High NLR: **Good pathologic response (Mandard grade 1–2)-**OR 0.53, 95% CI (0.26–1.02), p = 0.06
			After CRT	N/A	2.5	N/A	Correlation: (-)	Correlation: (-)
Lino-Silva et al.[26]	2016/Mexico	175 (100)	Before CRT	2.65 ± 1.32* (0.58–6.89)	3.0	17.7%	Correlation: (-) Low NLR vs. High NLR: **OS**—77.6% vs. 75.9%, p = 0.548	Correlation: (-) Low NLR vs. High NLR: **pCR—**no difference
Shen J et al.[27]	2017/China	202 (100)	Before CRT	2.4 (0.6–12.8)	3.0	31.2%	Correlation: (-) Low NLR vs. High NLR: **OS**—HR 1.066, 95% CI (0.681–1.668), p = 0.779 **DFS**—HR 0.863, 95% CI (0.536–1.390), p = 0.542	Correlation: (-) Low NLR vs. High NLR: **Good pathologic response (Dworak grade 3–4)** - 56.8% vs. 63.4%, p = 0.359
Jung et al.[28]	2017/Korea	984 (100)	Before CRT	N/A	1.7	55.5%	Correlation: (-) Low NLR vs. High NLR: **Recurrence free survival**—HR 1.319, 95% CI(0.978–5.087), p = 0.07	Correlation: (-) **TRG**—p = 0.25
Portale et al.[29]	2018/Italy	152 (32.2)	Before CRT or surgery	2.2 (IQR, 1.7–3.1)	N/A	N/A	Correlation: (-) NLR—Poor discriminative performance: **OS**—AUC 0.47 **DFS**—AUC 0.47	Not evaluated
This study	Korea	131 (100)	Before CRT	2.25 (IQR, 1.64–3.29)	3.0	29.8%	Correlation: (+) Control vs. pre-NLR< 3 & post-NLR< 3: **DFS**—HR 0.37, 95% CI (0.15–0.92), ***p = 0*.*033***	Correlation: (-) pre-NLR< 3 & post-NLR < 3 vs. control: **pCR—**21.3% vs. 14.9%, p = 0.593
			After CRT	3.46 (IQR, 2.57–4.75)	3.0	60.3%		

Abbreviations: OS: Overall survival, DFS: Disease-free survival, RFS: Relapse free survival, pCR: pathologic complete response, TRG: Tumor regression grade N/A: Not available

*: Mean ± SD, IQR: interquartile range

Although various studies showed the prognostic impact of NLR on survival, there was no consensus for cut-off values of NLR in patients with colorectal cancer. Some authors used an ROC curve to define the optimal cut-off value in which the variable of interest was death or pathologic tumor response.[16,19,25] Other studies decided on a point that could maximize survival differences.[21,28,32] Our cut-off value 3 was also arbitrary and was based on what had been studied or suggested in previous studies on colorectal cancers.[33–35] Nevertheless, the discriminative power of our cut-off value was well demonstrated in various patients with colorectal cancer irrespective of race, which makes our findings more generalizable.

When we divided our cohort into the persistently lower NLR group (group A) versus groups B-D, there was an uneven distribution of baseline characteristics, especially for clinical staging. The persistently lower NLR group (group A) was associated with earlier clinical T staging. Association of high NLR with advanced staging was also observed in other studies. Shen and colleagues reported a significantly higher clinical stage III rate in the high NLR group (95.4%) compared to that of the low NLR group (82.7%) (p = 0.012).[16] In another large-scale study evaluating various blood-derived immune parameters, patients in the high NLR group, which was defined as > 1.7 NLR, had more advanced ypT stage and ypN1 stage.[28] Based on these observations, we cannot exclude the possibility that NLR is a biomarker reflecting an advanced stage. Although the inherent skewness of clinical staging might translate into favorable or poor survival outcomes, we tried to balance clinical staging between the comparative groups using propensity score matching. One strength of this study is the effort to reduce selection bias.

When NLR values measured before starting preop-CRT and after completion of preop-CRT in previous studies were compared (Table 4), the median NLR value measured after completion of CRT is relatively higher than that measured before initiation of CRT (2.4→3.7 in Caputo et al., 2.1→3.23 in Sung et al. respectively).[24,32] In the same sense, some studies reported that the rate of high NLR increased after completion of CRT (24.5%→49% in Kim IY et al., 9.6%→ 27.1% in Lee et al. respectively).[18,25] This phenomenon was also observed in our study. Lee and colleagues reported that neutrophil and lymphocyte counts were maximally decreased 2 weeks after CRT onset in patients with rectal cancer.[30] The neutrophil count increased slightly until the date of surgery; however, the lymphocyte count further decreased until 1 month after commencing preop-CRT. Similarly, Kitayama and colleagues reported that the numbers of neutrophils and monocytes were comparably maintained, while circulating lymphocytes were most markedly decreased during CRT for patients with rectal cancer.[38] Based on these observations, it seems reasonable to assume that decreased peripheral lymphocytes may be one of the major reasons for increased NLR value after completion of preop-CRT. Lymphocytes are the most radio-sensitive cells, and lymphocyte LD_50_ (lethal dose 50) is among the lowest in the body.[39,40] Reasons for depletion of peripheral lymphocyte after radiation therapy have been suggested as direct irradiation of circulating lymphocytes and/or bone marrow suppression, although the causes of this hematologic response are diverse and remain unknown for rectal cancer.[41]

The physiologic role of lymphocytes is to suppress tumor progression via cytotoxic cell death and cancer immune surveillance.[42] The association of radiation-induced lymphopenia with poor survival has been reported in various solid tumors including brain tumors, head and neck cancer, lung cancer, esophageal cancer, breast cancer, pancreatic cancer, and cervical cancer.[41] The clinical impact of lymphocyte count during preop-CRT in patients with rectal cancer was investigated to show that sustaining lymphocyte ratio ≥ 0.35 at 4 weeks after commencement of CRT, which was defined as lymphocyte count at 4 weeks divided by baseline lymphocyte count, was an independent predictor of pCR.[43] However, the host response on depletion and/or recovery of circulating lymphocytes during radiation therapy might be different. In our group, among patients who showed pre-NLR <3, 48.9% of patients were categorized into the >3 post-NLR group, while 51.1% of patients stayed in the <3 post-NLR group. The current study could not definitively explain why this kind of hematologic response was different among patients, although the explanation is likely multifactorial. In brief, our results suggest that the preserved host immunity identified during the preop-CRT period can be retained as a long-term effect for patients with rectal cancer, although further studies are needed to validate our findings.

As part of further analyses, we evaluated that the number of neutrophil, lymphocytes, PLR and LMR had some prognostic impact. When we dichotomized our patients into the two separate groups using the median value of pre- and post- measurements, there was no survival difference between the two groups (S1 and S2 Tables). These trends lasted when we sub-divided patients in more detail (S1A–S1D Fig, S3 Table). Some previous studies demonstrated that PLR or LMR had prognostic power in rectal cancer patients who underwent preop-CRT.[28,44–47] Nevertheless, there were some contradictory results[20,48,49] and the cut-off points were heterogeneous among the studies.[44–47] Although our study did not reveal any association between PLR and LMR with survival outcomes, the small sample size of our study and unconfirmed cut-off values might hinder to make a concrete conclusion. It was demonstrated that NLR, PLR and LMR were basically positively correlated with each other.[28,50] Thus, comparing the prognostic impact of PLR, LMR or NLR should be thoroughly investigated and discussed with large populations to exclude the possible interaction between these parameters.

Some limitations of this study have to be acknowledged. The main limitation is derived from the retrospective and single center-based study design. Thus, critical postoperative pathologic outcomes, such as circumferential resection margin, lymphovascular invasion, and perineural invasion, were not adequately included in final survival analysis. The low specificity of NLR is an inherent limitation of this design. Various conditions, such as hidden infection or pharmacologic response, might influence the value of NLR. Although our study tried to exclude patients with specific conditions such as inflammatory bowel disease-associated rectal cancer, the inherent retrospective nature of this study could not fully exclude patients who had undetectable external sources of inflammatory reaction when blood was sampled. Although the cut-off value used in our study was suggested by previous studies, diverse cut-off values are used in different studies (Table 4). The racial difference of normal NLR values might make it more difficult to generate a universal reference.[6,36,37] The lack of consensus on cut-off values remains a serious problem and hampers clinical utilization of these findings.

In conclusion, our study demonstrated that persistent lower NLRs during preop-CRT is associated with low possibility of recurrence after surgery in patients with non-metastatic rectal cancer. Considering the debate on the necessity of adjuvant chemotherapy for patients with rectal cancer after preop-CRT, especially for patients with good tumor response,[51,52] our observation might be used to stratify patients for additional treatments. Further studies to validate this hypothesis are needed.

## Supporting information

S1 Dataset Raw data of 94 patients after PSM. (XLSX)

S1 Fig OS and DFS according to the combination of pre and post PLRs and LMRs (n = 94). (DOCX)

S1 Table Median and interquartile ranges of neutrophil, lymphocyte, PLR and LMR measured in pre and post CRT (n = 94). (DOCX)

S2 Table Univariable analysis of OS and DFS according to neutrophil, lymphocyte, PLR and LMR of pre and post measurements (n = 94). (DOCX)

S3 Table Univariable analysis of OS and DFS according to the combination of pre and post PLRs and LMRs (n = 94). (DOCX)
