# Supplementary material for: Prognostic impact of persistent lower neutrophil-to-lymphocyte ratio during preoperative chemoradiotherapy in locally advanced rectal cancer patients: A propensity score matching analysis
Source: PLoS One. 2019 Mar 22;14(3):e0214415. doi: 10.1371/journal.pone.0214415 (PMC6430363; doi:10.1371/journal.pone.0214415)
Supplement: S1 Fig — (DOCX) [file pone.0214415.s002.docx]

S1 Figure. OS and DFS according to the combination of pre and post PLRs and LMRs (n=94).

| A) OS according to the 4 subgroups via combination of pre and post PLRs | B) DFS according to the 4 subgroups via combination of pre and post PLRs |
| --- | --- |
| p = 0.628 | p = 0.396 |
| 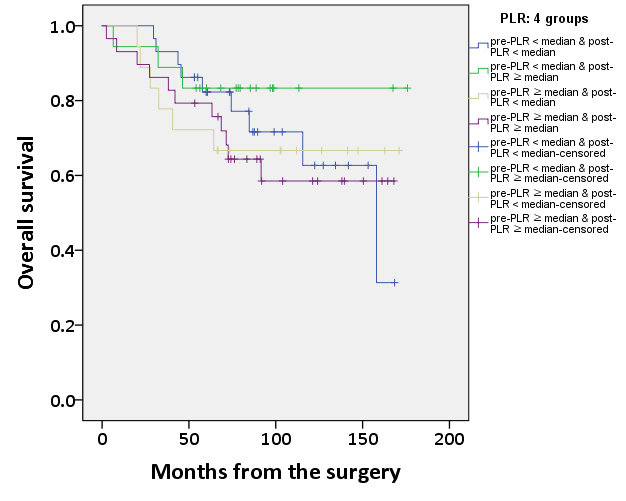 | 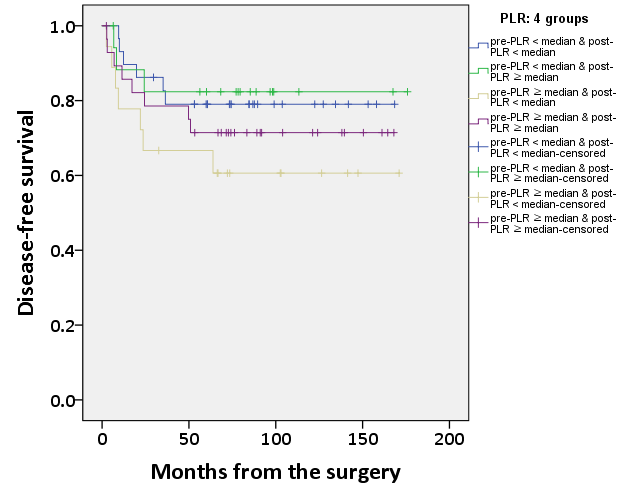 |
|  |  |
| C) OS according to the 4 subgroups via combination of pre and post LMRs | D) DFS according to the 4 subgroups via combination of pre and post LMRs |
| p = 0.970 | p = 0.796 |
| 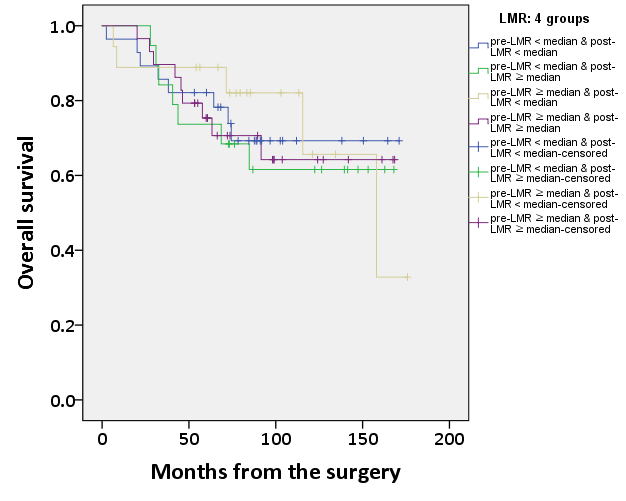 | 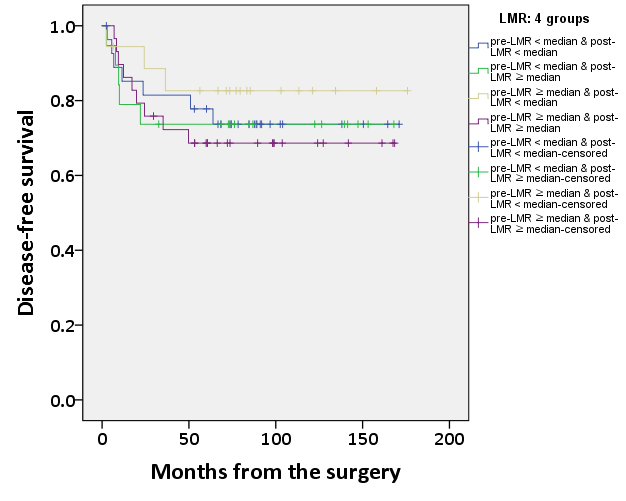 |
