# Supplementary material for: Prognostic impact of persistent lower neutrophil-to-lymphocyte ratio during preoperative chemoradiotherapy in locally advanced rectal cancer patients: A propensity score matching analysis
Source: PLoS One. 2019 Mar 22;14(3):e0214415. doi: 10.1371/journal.pone.0214415 (PMC6430363; doi:10.1371/journal.pone.0214415)
Supplement: S1 Table — (DOCX) [file pone.0214415.s003.docx]

Supporting information

**Prognostic impact of persistent lower neutrophil-to-lymphocyte ratio during preoperative chemoradiotherapy in locally advanced rectal cancer patients: A propensity score matching analysis**

S1 Table. Median and interquartile ranges of neutrophil, lymphocyte, PLR and LMR measured in pre and post CRT (n=94).

| Variables |  |  |
| --- | --- | --- |
| pre-neutrophil (10^3^/㎕) | median (IQR) | 3.61 (2.82 – 4.51) |
| post-neutrophil (10^3^/㎕) | median (IQR) | 2.95 (2.11 – 3.81) |
| pre-lymphocyte (10^3^/㎕) | median (IQR) | 1.78 (1.31 – 2.2) |
| post-lymphocyte (10^3^/㎕) | median (IQR) | 0.96 (0.73 – 1.15) |
| pre-PLR | median (IQR) | 154.4 (126.5 – 193.8) |
| post-PLR | median (IQR) | 255.7 (199.5 – 348.7) |
| pre-LMR | median (IQR) | 5.42 (4.07 – 6.97) |
| post-LMR | median (IQR) | 3.15 (2.39 – 4.16) |

Abbreviations; PLR: platelet to lymphocyte ratio; LMR: lymphocyte to monocyte ratio

IQR: interquartile range
