# Supplementary material for: Prognostic impact of persistent lower neutrophil-to-lymphocyte ratio during preoperative chemoradiotherapy in locally advanced rectal cancer patients: A propensity score matching analysis
Source: PLoS One. 2019 Mar 22;14(3):e0214415. doi: 10.1371/journal.pone.0214415 (PMC6430363; doi:10.1371/journal.pone.0214415)
Supplement: S2 Table — (DOCX) [file pone.0214415.s004.docx]

S2 Table. Univariable analysis of OS and DFS according to neutrophil, lymphocyte, PLR and LMR of pre and post measurements (n=94).

|  | OS |  | DFS |  |
| --- | --- | --- | --- | --- |
|  | Hazard Ratio (95% CI) | P | Hazard Ratio (95% CI) | P |
| pre-neutrophil |  |  |  |  |
| ≥ 3.61 vs. < 3.61 | 0.95 (0.45 – 1.98) | 0.893 | 1.21 (0.54 – 2.74) | 0.633 |
| post-neutrophil |  |  |  |  |
| ≥ 2.95 vs. < 2.95 | 1.34 (0.64 – 2.82) | 0.428 | 1.71 (0.75 – 3.92) | 0.199 |
| pre-lymphocyte |  |  |  |  |
| ≥ 1.78 vs. < 1.78 | 0.81 (0.39 – 1.70) | 0.589 | 0.64 (0.28 – 1.46) | 0.296 |
| post-lymphocyte |  |  |  |  |
| ≥ 0.96 vs. < 0.96 | 0.88 (0.42 – 1.82) | 0.732 | 0.88 (0.39 – 1.96) | 0.760 |
| pre-PLR |  |  |  |  |
| ≥ 154.4 vs. < 154.4 | 1.46 (0.69 – 3.06) | 0.313 | 1.82 (0.79 – 4.15) | 0.156 |
| post-PLR |  |  |  |  |
| ≥ 255.7 vs. < 255.7 | 0.93 (0.45 – 1.94) | 0.864 | 0.86 (0.38 – 1.92) | 0.713 |
| pre-LMR |  |  |  |  |
| ≥ 5.42 vs. < 5.42 | 0.95 (0.46 – 1.97) | 0.898 | 0.96 (0.43 – 2.15) | 0.937 |
| post-LMR |  |  |  |  |
| ≥ 3.15 vs. < 3.15 | 1.18 (0.56 – 2.46) | 0.653 | 1.37 (0.61 – 3.09) | 0.443 |

Abbreviations; PLR: platelet to lymphocyte ratio; LMR: lymphocyte to monocyte ratio
