# Supplementary material for: Prognostic impact of persistent lower neutrophil-to-lymphocyte ratio during preoperative chemoradiotherapy in locally advanced rectal cancer patients: A propensity score matching analysis
Source: PLoS One. 2019 Mar 22;14(3):e0214415. doi: 10.1371/journal.pone.0214415 (PMC6430363; doi:10.1371/journal.pone.0214415)
Supplement: S3 Table — (DOCX) [file pone.0214415.s005.docx]

S3 Table. Univariable analysis of OS and DFS according to the combination of pre and post PLRs and LMRs (n=94).

|  | OS |  | DFS |  |
| --- | --- | --- | --- | --- |
|  | Hazard Ratio  (95% CI) | P | Hazard Ratio  (95% CI) | P |
| Combination of pre&post PLRs |  |  |  |  |
| pre-PLR<154.4 & post-PLR<255.7 vs. control | 0.97 (0.44 – 2.13) | 0.941 | 1.47 (0.58 – 3.71) | 0.412 |
| Combination of pre&post LMRs |  |  |  |  |
| pre-LMR<5.42 & post-LMR<3.15 vs. control | 1.18 (0.56 – 2.46) | 0.653 | 1.37 (0.61 – 3.09) | 0.443 |
| pre-LMR≥5.42 & post-LMR≥3.15 vs. control | 0.93 (0.42 – 2.05) | 0.862 | 0.74 (0.32 – 1.73) | 0.485 |
